# The integration of AlphaFold-predicted and crystal structures of human *trans*-3-hydroxy-l-proline dehydratase reveals a regulatory catalytic mechanism

**DOI:** 10.1016/j.csbj.2022.07.027

**Published:** 2022-07-18

**Authors:** Eugenio Ferrario, Riccardo Miggiano, Menico Rizzi, Davide M. Ferraris

**Affiliations:** a University of Piemonte Orientale, Department of Pharmaceutical Sciences, Largo Donegani 2, 28100 Novara, Italy; b IXTAL srl, Via Bovio 6, 28100 Novara, Italy

**Keywords:** Computational protein structure prediction, Computational oligomerization prediction, AlphaFold, Crystal structure, *Trans*-3-Hydroxy-l-proline, Dehydratase

## Abstract

Computational methods for protein structure prediction have made significant strides forward, as evidenced by the last development of the neural network AlphaFold, which outperformed the CASP14 competitors by consistently predicting the structure of target proteins. Here we show an integrated structural investigation that combines the AlphaFold and crystal structures of human *trans*-3-Hydroxy-l-proline dehydratase, an enzyme involved in hydroxyproline catabolism and whose structure had never been reported before, identifying a structural element, absent in the AlphaFold model but present in the crystal structure, that was subsequently proved to be functionally relevant. Although the AlphaFold model lacked information on protein oligomerization, the native dimer was reconstructed using template-based and *ab initio* computational approaches. Moreover, molecular phasing of the diffraction data using the AlphaFold model resulted in dimer reconstruction and straightforward structure solution. Our work adds to the integration of AlphaFold with experimental structural and functional data for protein analysis, crystallographic phasing and structure solution.

## Graphical abstract

**Figure:**
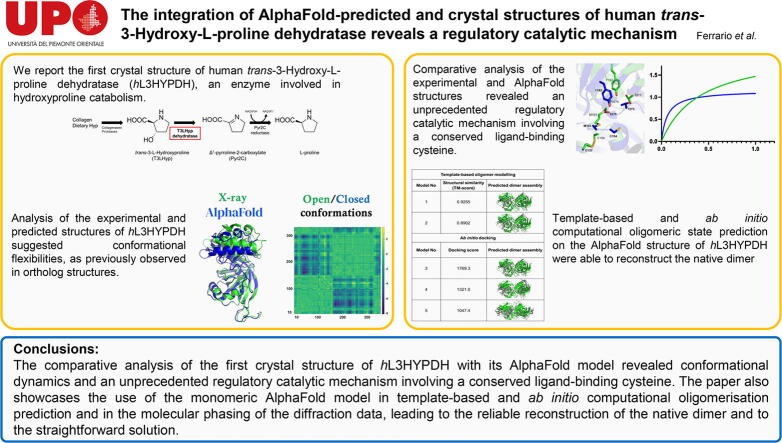

## 1 Introduction

Since 1994, the Critical Assessment of protein Structure Prediction (CASP) [1] represents the biennial event in which worldwide research groups showcase their protein structure prediction mastery by competing for the solution of unsolved protein structures, leading to the development of increasingly reliable computational methods for structure prediction and validation. More recently, the advent of artificial intelligence and the use of neural networks allowed an unparalleled accuracy of the predicted structural model, which saw its culmination in CASP14 [2], where AlphaFold2, the latest version of the AlphaFold (AF) program [3], outperformed the competitors by accurately and regularly solving protein structures, even in absence of a structural homolog [4], [5]. This remarkable achievement has impacted the scientific community by predicting the structures of nearly 98.5 % of the human proteome [4], [6], with the ambition of tackling the proteomes of other organisms in the future. Hence, the AF database provides a gold mine of reliable, computationally predicted protein models awaiting experimental structure solution, that still account for nearly 80 % of the human proteome [7].

One of the challenges in structural biology is the exploitation and the harmonization of the plethora of data derived from computational and multiple experimental sources, and the emerging field of integrative structural biology aims at combining predictive computational methods with still unresolved experimental structural data [8]. In this framework, we have focused our attention on human *trans*-3-Hydroxy-l-proline dehydratase (*h*L3HYPDH), an enzyme for which the reports concerning its function and structure are scant or absent, thus representing a suitable target for integrating predictive and experimental data for advancing the knowledge over its structure and function.

**Fig. 1:**
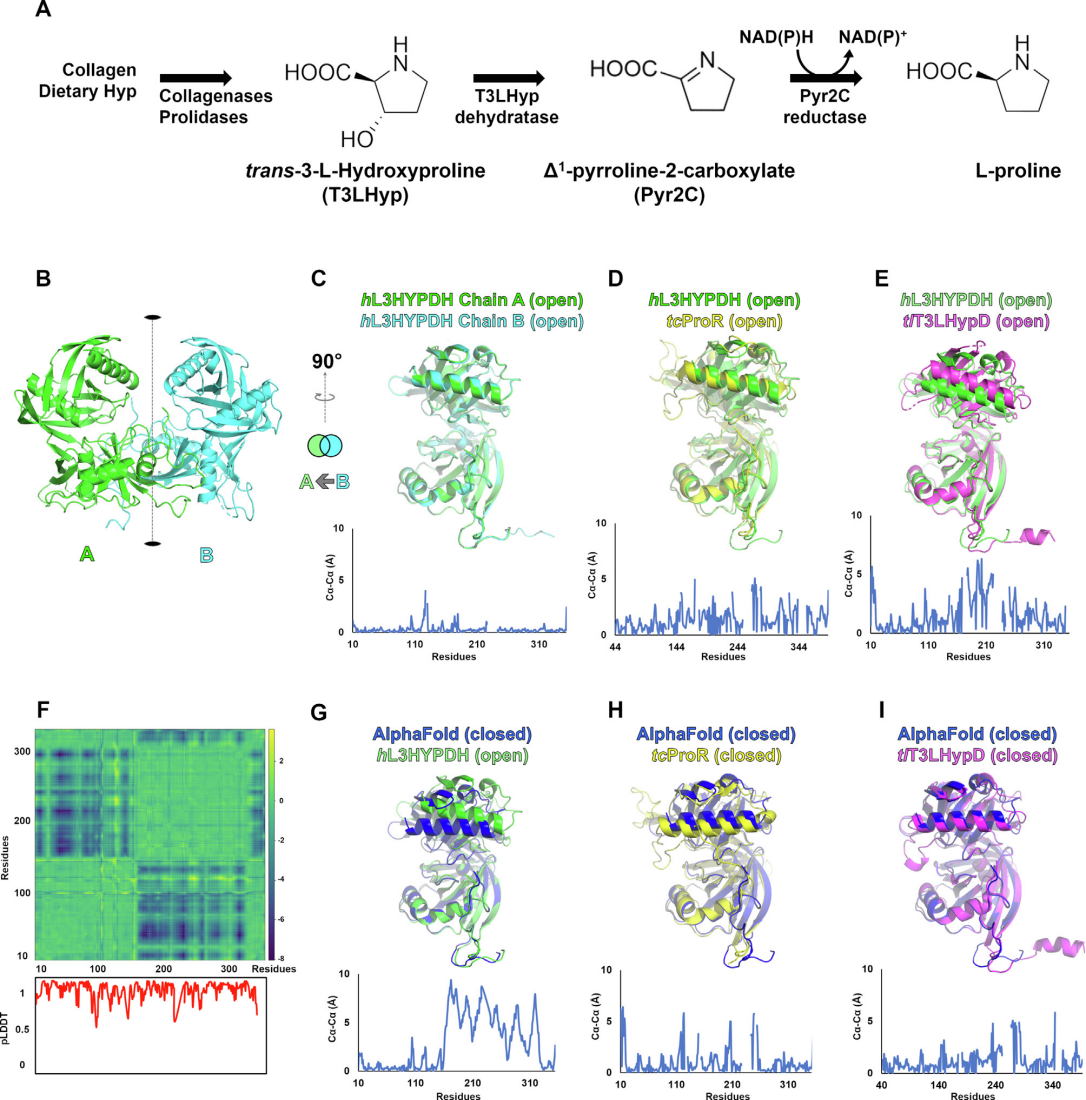
Reaction scheme of *trans*-3-Hydroxy-l-proline (T3LHyp) metabolism in humans and conformational analysis of predicted and experimental *h*L3HYPDH structures with protein homologs. A) Collagen and dietary T3LHyp is degraded by collagenases and prolidases forming free T3LHyp, which is then processed by T3LHyp dehydratase to form Δ^1^-pyrroline-2-carboxylate (Pyr2C). The last metabolic step involves the NAD(P)H-dependent Pyr2C reductase which converts Pyr2C into l-proline. B) Experimental structure of *h*L3HYPDH: chains A and B are shown in green and cyan, respectively, and the non-crystallographic twofold vertical axis relating the two chains is shown as a dotted line. C) Superposition of experimental *h*L3HYPDH chain B over chain A and 90° anticlockwise rotation. D) Superposition of *tc*ProR open monomer (in yellow) over chain A of experimental *h*L3HYPDH (in green); E) Superposition of *tl*T3LHypD open monomer (in magenta) over chain A of experimental *h*L3HYPDH (in green). F) Distance-difference matrix between equivalent Cα atoms of the experimental and predicted *h*L3HYPDH models. Blue-green colouring indicates changes in the Cα distances, with blue colour indicating the most distant, and the green colour indicating the closest. Below, per-residue confidence score (pLDDT) of predicted *h*L3HYPDH as calculated by AF. G) Superposition of the experimental open and the AF closed structures of *h*L3HYPDH (in green and in blue, respectively); H) Structural alignment between *tc*ProR in the closed conformation (in yellow) and the AF closed *h*L3HYPDH structure (in blue); I) Superposition of *tl*T3LHypD in the closed conformation (in magenta) over the AF closed *h*L3HYPDH structure (in blue). Conformational analysis was carried out superimposing the homolog structures against the dimerization domain of *h*L3HYPDH structures (residues 10–149), thus excluding the mobile domains from the structural alignment and highlighting their conformational differences. Structure divergence plots were calculated using the PyMod 3 suite [24]. Blue-line graphs represent the structure divergence plots between the corresponding aligned chains, indicating on the abscissa the residues numbers and on the ordinate the Cα distances expressed in Å. (For interpretation of the references to colour in this figure legend, the reader is referred to the web version of this article.)

*h*L3HYPDH is involved in the metabolism of hydroxyproline (Hyp), a non-standard amino acid present in the cell wall components of plants [9] and in mammalian collagen [10], [11] and deriving from the post-translational modification of proteins by prolyl hydroxylase enzymes [12]. Some plants and bacteria produce Hyp, and the isomers *trans*-3-Hydroxy-l-proline (T3LHyp) and *trans*-4-Hydroxy-l-proline (T4LHyp) are major components of mammalian collagen. While T4LHyp is metabolised following distinct degradative pathways in mammals and bacteria [13], the T3LHyp metabolic pathway is conserved in bacteria, plants and mammals, and involves a T3LHyp dehydratase (EC 4.2.1.77) which removes the hydroxyl group of T3LHyp without the intervention of a cofactor, leading to the formation of Δ^2^-pyrroline-2-carboxylate (Fig. 1A). This reaction product spontaneously converts into Δ^1^-pyrroline-2-carboxylate (Pyr2C) and is then transformed in l-proline by a NAD(P)H-dependent Pyr2C reductase (EC 1.5.1.21) [14] which removes the double bond from the pyrroline intermediate (Fig. 1A).

*h*L3HYPDH was first discovered by Visser and colleagues [15] who identified, through sequence alignments between orthologs of the proline racemases family, the human protein C14orf149 (named after its gene locus and later named *h*L3HYPDH) which lacked racemase activity but exhibited instead proline dehydratase activity, converting *trans*-3-hydroxy-l-proline (T3LHyp) into Δ^1^-pyrroline-2-carboxylate (Pyr2C). Besides its role in the dietary hydroxyproline metabolism, *h*L3HYPDH has been also identified among the interferon-stimulated genes (ISGs) triggered by virus infection and showing antiviral activities [16], [17], [18]. More recently, *h*L3HYPDH has been associated with the genetic regulation of the working memory [19] and has been also observed that the *h*L3HYPDH-coding gene is differentially methylated in the mitochondrial pathway involved in autism spectrum disorder associated with Glutaryl-CoA degradation [20]. As of to date, no structural data of *h*L3HYPDH have been reported, making it a suitable target for stressing the predictive power of AF in the *de novo* structure solution.

In the context of advancing structural biology by integrating the AF structures with experimental data, here we show the first crystal structure of *h*L3HYPDH and the comparative analysis with its AF model [21], revealing conformational dynamics and an unprecedented regulatory catalytic mechanism involving a conserved ligand-binding cysteine. We also show the use of the monomeric AF model in template-based and *ab initio* computational oligomerisation prediction and in the molecular phasing of the diffraction data, leading to the reliable reconstruction of the native dimer and to the straightforward solution of the native structure of *h*L3HYPDH.

## 2 Results

### 2.1 Determination of the experimental structure of hL3HYPDH and conformational analysis.

Crystal screening and optimization of recombinant *h*L3HYPDH in absence and in presence of the substrate or the transition-state analogue pyrrole-2-carboxylic acid (PYC) produced crystals that best diffracted at 3.0 Å (Table 1). The final *h*L3HYPDH model was reliably built between amino acids 10–354 (for chain A) and 4–354 (for chain B) except for residues 150–152 and 227–239 of both chains due to the missing or poor-quality electron density. The *h*L3HYPDH structure consists of an α/β dimeric protein recapitulating the structure of the orthologs *T. litoralis trans*-3-hydroxy-l-proline dehydratase (*tl*T3LHypD; PDB code: 6R76 and 6R77; 48 % identity) [22] and *T. cruzi* proline racemase (*tc*ProR; PDB codes: 1 W61 and 1 W62; 37 % identity) [23], with a root-mean square deviation (RMSD) of 1.29 Å and 1.10 Å between equivalent Cα, respectively. A dimerization domain (residues 10–149) and a mobile, jaw-like domain (residues 153–332) topping the previous complete the catalytic sites of the two *h*L3HYPDH monomers (Fig. 1B).

**Table 1:** Data collection and refinement statistics.

Wavelength (Å)	0.9686
Resolution range (Å)	44.98–3.0 (3.1–3.0)
Space group	P 2_1_ 2_1_ 2
Unit cell parameters	a = 114.05b = 122.92c = 73.16
a, b, c (Å) α, β, γ (°)	α = 90.0° β = 90° γ = 90.0°
Total reflections	39,669 (3930)
Unique reflections	20,658 (2049)
Multiplicity	1.9 (1.9)
Completeness (%)	96.90 (98.89)
Mean I/sigma(I)	5.57 (1.56)
Wilson B-factor (Å^2^)	76.83
R-merge	0.08158 (0.4657)
R-meas	0.1154 (0.6586)
R-work	0.22 (0.32)
R-free	0.25 (0.35)
RMS (bonds) (Å)	0.011
RMS (angles) (°)	1.30
Ramachandran favoured (%)	96.3
Ramachandran allowed (%)	3.67
Ramachandran outliers (%)	0.00
Rotamer outliers (%)	0.00
Clash score	5.78
Average B-factor (Å^2^)	76.8

Statistics for the highest-resolution shell are shown in parentheses.

Although *h*L3HYPDH crystallized in presence of the substrate T3LHyp or the transition state analogue PYC, examination of the catalytic centres of the two monomers did not reveal electron density attributable to these molecules. Unlike the structures of *tl*T3LHypD and of *tc*ProR that both showed a closed conformation for the ligand-complexed monomer and an open conformation of the ligand-free monomer [22], [23], both *h*L3HYPDH monomers exhibited an open conformation, consistent with the absence of ligands in both catalytic sites. Indeed, the structural and conformational match of the two open monomers of *h*L3HYPDH was confirmed by structural alignment and structure divergence plot [Root-mean-square deviation (RMSD) of 0.239; Fig. 1C].

Comparative conformational analysis of *h*L3HYPDH with homolog structures showed that the conformation of the *h*L3HYPDH mobile domain best matched that of the open monomer of *tc*ProR (RMSD = 1.095 Å; Fig. 1D), showing also a more restrained movement compared to the ligand-free, open *tl*T3LHypD structure, which presents a wider opening instead (Fig. 1E).

### 2.2 Comparative analysis of the experimental and AlphaFold structures of *h*L3HYPDH.

The predicted AF model consists in a monomeric domain that faithfully recapitulates the α/β folding and overall architecture of the experimental *h*L3HYPDH structure. However, structural alignment between the predicted and experimental *h*L3HYPDH models revealed a significant difference in the conformation of the mobile domains, being the experimental and the predicted structure in the open and closed conformation, respectively. Such difference is emphasized by the distance difference matrix and by the structure divergence plot (Fig. 1F and 1G and Supplementary Video 1 and 2) which qualitatively and quantitatively show the conformational differences between the open (experimental) and the closed (predicted) *h*L3HYPDH structures.

The predicted closed structure of *h*L3HYPDH was compared to the closed monomers of *tc*ProR (Fig. 1H) and *tl*T3LHypD (Fig. 1I), revealing conformational similarities between the *h*L3HYPDH AF model and the closed conformations of *tc*ProR and *tl*T3LHypD, the latter being the most conformationally related (RMSD = 2.02 Å and 1.10 Å, respectively).

### 2.3 Analysis of the molecular determinants of *h*L3HYPDH conformational dynamics, catalysis, and regulation

**Fig. 2:**
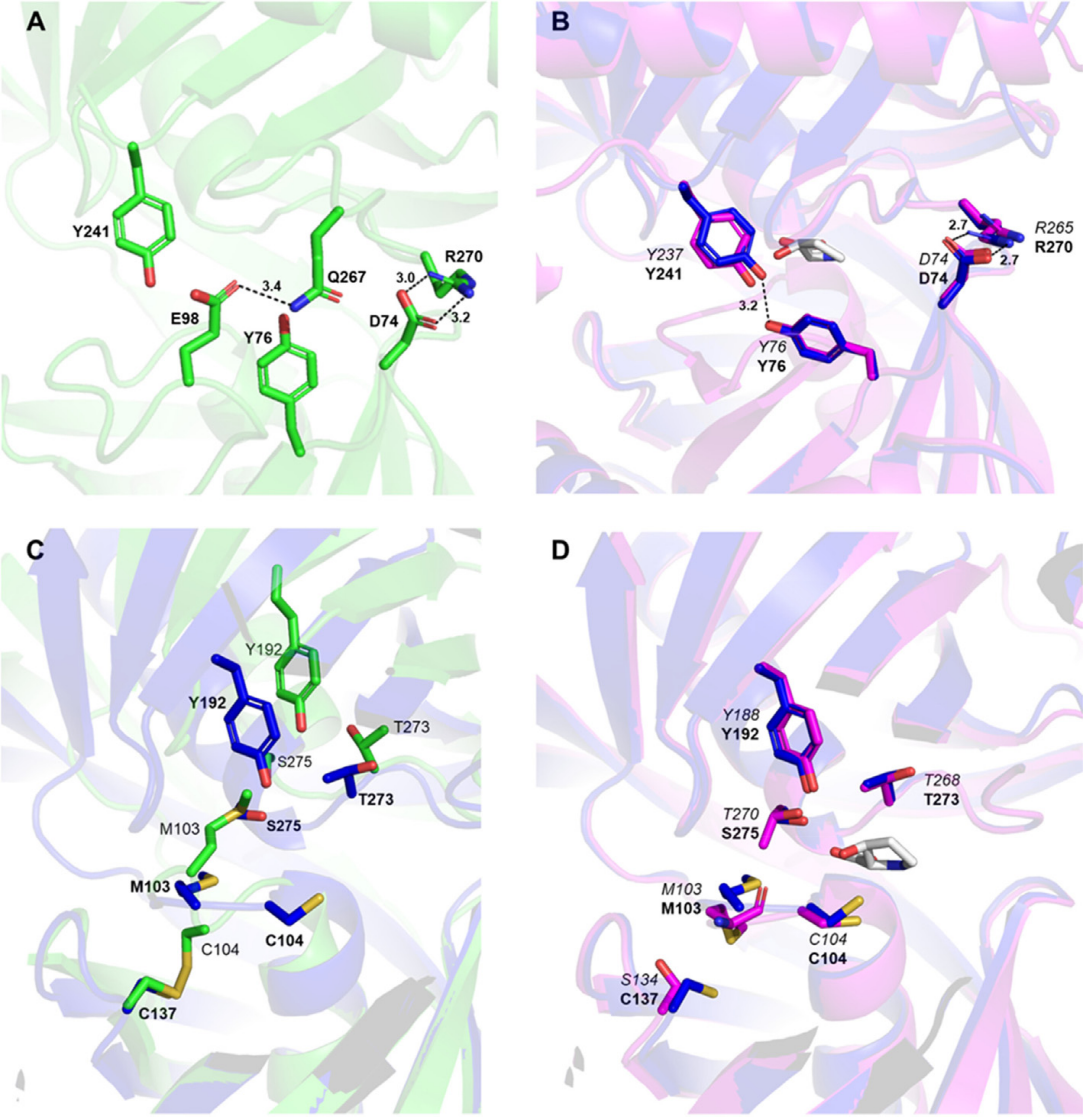
Molecular determinants of domain mobility and catalysis. A) Molecular interactions stabilizing the *h*L3HYPDH open conformations. In green sticks and ribbons, the experimental open *h*L3HYPDH structure. B) Molecular interactions stabilizing the *h*L3HYPDH closed conformation (in blue sticks and ribbons and in bold letters, the computational *h*L3HYPDH closed structure; in magenta sticks and ribbons and in italics, the closed structure of *tl*T3LHypD; PDB code: 6R77). The substrate T3LHyp is shown in grey. C) Superposition of the catalytic sites of the experimental open *h*L3HYPDH structure (in green sticks and ribbons and in bold letters) with the predicted *h*L3HYPDH structure (closed conformation; in blue sticks and ribbons). The intramolecular disulphide bond occurring between Cys104 and Cys137 in the experimental *h*L3HYPDH structure is shown. This interaction is lost in the predicted *h*L3HYPDH structure. D) Superposition of the catalytic sites of the predicted *h*L3HYPDH structure (closed conformation; blue sticks and ribbons and in bold letters) with the *tl*T3LHypD structure in the closed conformation (PDB code: 6R77; in magenta sticks and ribbons and in italics). (For interpretation of the references to colour in this figure legend, the reader is referred to the web version of this article.)

The experimental open and the predicted closed structures of *h*L3HYPDH allowed us to examine the molecular interactions stabilizing the two conformations. A common feature observed in both structures is the salt bridge between residues D74 and R270 that varies in distance from an average of 3.1 Å in the experimental open state to 2.7 Å in the computational closed conformation (Fig. 2A and 2B). Notably, residues D74 and R270 are also conserved in *tc*ProR and *tl*T3LHypD (Fig. 4) and in the proline racemase enzyme family [15], thus highlighting their importance in the stabilization of the open and closed conformations. Moreover, a hydrogen bond between Asp98 and Gln267 (3.4 Å) further stabilizes the open conformation (Fig. 2A); however, this interaction is lost in the closed state, as observed in the predicted structure, where Tyr76 and Tyr241 engage in an H-bond (3.2 Å; Fig. 2B). Hence, experimental and computational analysis suggest that the Asp98-Gln267 and Tyr76-Tyr241 residue pairs play complimentary roles in the stabilization of the open and closed conformations of *h*L3HYPDH (see Supplementary Video 3).

**Fig. 3:**
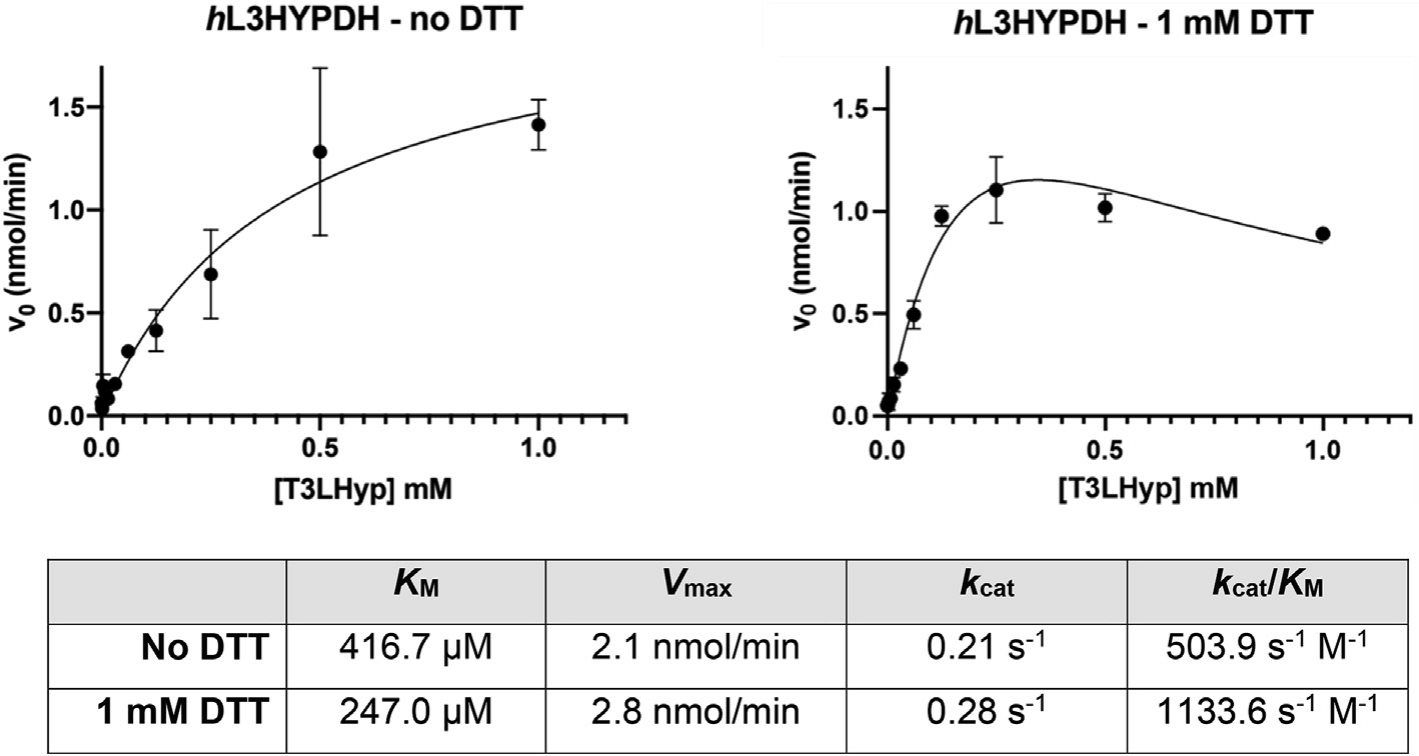
*h*L3HYPDH catalytic parameters under oxidizing and reducing conditions. Michaelis-Menten curves of *h*L3HYPDH measured in absence (left) and presence (right) of 1 mM DTT. The table below reports the Michaelis-Menten parameters measured under oxidizing and reducing condition.

**Fig. 4:**
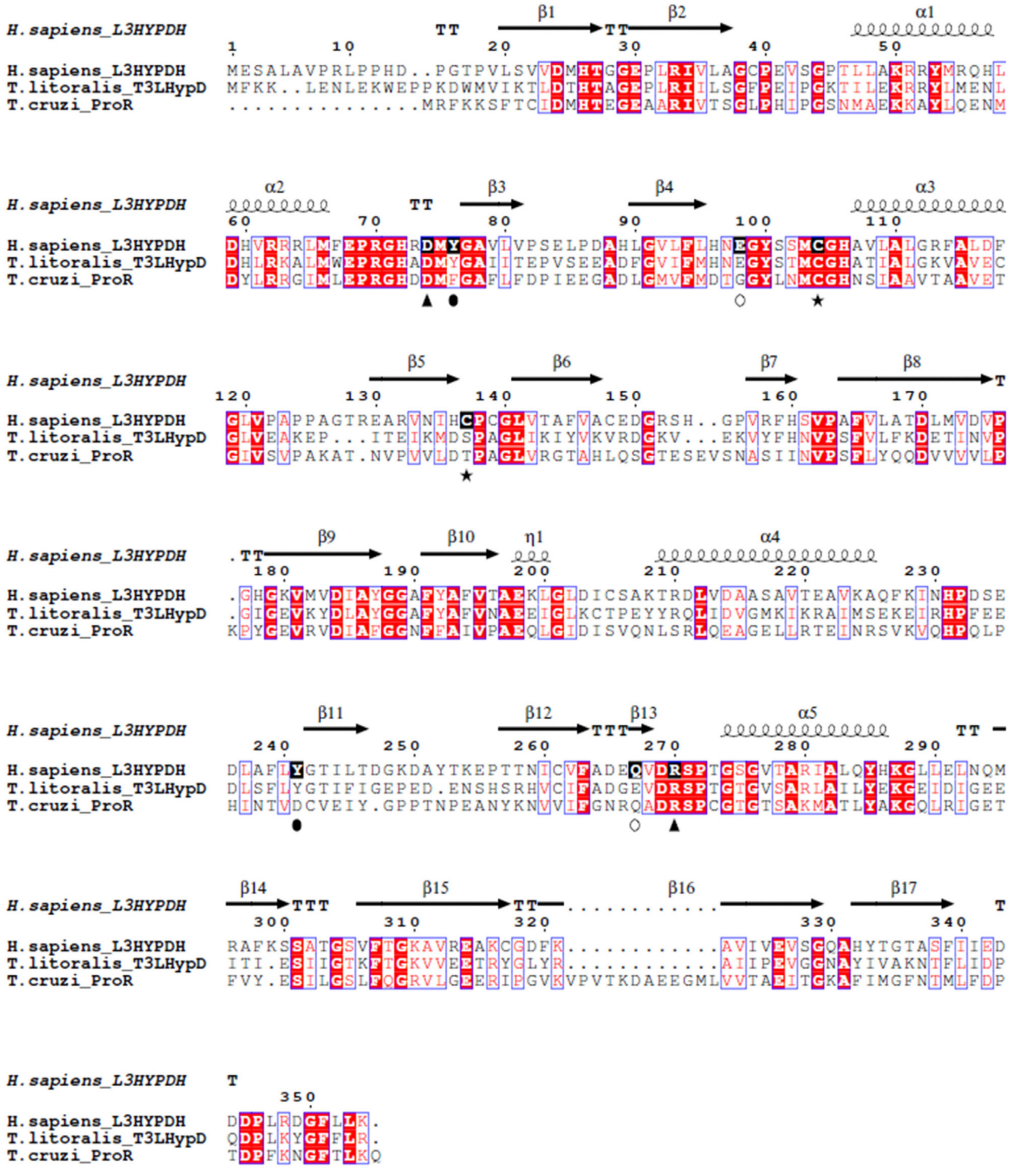
Sequence alignments of human *h*L3HYPDH with homolog proteins. Sequence alignments of human *h*L3HYPDH with *T. litoralis* T3LHypD (*tl*T3LHypD; 48% identity) and *T. cruzi* ProR proline racemase (*tc*ProR; 37% identity). Spirals and arrows indicate α-helices and β-strands of *h*L3HYPDH, respectively. Red boxes with white characters indicate residue identity; red characters indicate residue similarity; blue-framed characters indicate similarities between groups of residues. All interacting amino acids described in the text and stabilizing the open and closed conformations of *h*L3HYPDH are boxed in black with white letters: Asp74 and Arg270 involved in the salt bridge in the closed conformation are labelled with a black triangle; Tyr76 and Tyr 241 engaged in hydrogen bonding in the closed conformation are labelled with a black oval. Asp98 and Asn267 forming a salt bridge in the open conformation are labelled with hollow circles. Cys104 and Cys137 forming the intramolecular disulphide bond in the experimental *h*L3HYPDH structure are labelled with a black star. Sequence alignments and editing was performed using Clustal Omega [25] and ESPript [26]. (For interpretation of the references to colour in this figure legend, the reader is referred to the web version of this article.)

The catalytic site of the predicted *h*L3HYPDH model (closed conformation, Fig. 2C) retains the general arrangement of the amino acids involved in ligand binding as in the ortholog protein *tl*T3LHypD, with residues Tyr192, Ser275, and Met103 of *h*L3HYPDH matching the *tl*T3LHypD catalytic triad composed by Tyr188, the conservatively mutated Thr270, and Met103 (Fig. 2D and Fig. 4). In *h*L3HYPDH, residues Met103 and Ser275 play a stabilizing role of the open conformation through hydrogen bonding of the hydroxy group of Ser275 and the sulphur of Met103 (3.0 Å), locking Met103 in an extended conformation and protruding it toward the free substrate-binding site (Fig. 2C and Supplementary Fig. 1 and Supplementary Video 4). Moreover, a distinct feature present in the experimental *h*L3HYPDH structure and absent in other homologous structures is observed for Cys104 which, together with Thr273, engages in substrate binding. In the experimental open structure, Cys104 is involved in an unprecedented intramolecular disulphide bond with the neighbouring Cys137 (Fig. 2C). This interaction, validated by crystallographic and mass spectrometry analysis (see Supplementary Figures 2-6 in Supplementary Material), suggested a sequestering mechanism and a catalytic regulatory role of Cys104. We investigated its catalytic role by measuring the *h*L3HYPDH activity under oxidizing and reducing conditions, i.e. in absence and presence of the reducing agent DTT, respectively. Experiments showed that the addition of 1 mM DTT reduced the *K*_M_ to 247.0 μM compared to the *K*_M_ of 416.7 µM measured without DTT, while maintaining substantially unaltered the *V*_max_ (Fig. 3). Hence, these findings point to a catalytic regulatory role for the intramolecular disulphide involving Cys104 and Cys137. Moreover, the kinetic data deviate from a canonical Michaelis-Menten curve, indicating substrate inhibition.

### 2.4 Analysis and oligomeric prediction of experimental and computational *h*L3HYPDH structures

Computational analysis of the dimer interface of the experimental *h*L3HYPDH structure showed a dimerization surface with a total interface area of 1914.3 Å^2^, more extended compared to the interface area of *tc*ProR (1471.4 Å^2^), but less extended to that of the thermophile *tl*T3LHypD (2640.1 Å^2^; Table 2). Although the experimental structures of *h*L3HYPDH and of its homologs show their dimeric nature, the predicted *h*L3HYPDH model lacked information regarding protein oligomerization.

**Table 2:** Comparative interface area analysis of experimental *h*L3HYPDH and computational oligomeric state predictions of the AF structure. The top table reports the interface and buried area analysis of the experimental dimeric structures of *h*L3HYPDH, *tc*ProR and *tl*T3LHypD. The analysis was performed using the COCOMAPS server [30]. Below are reported the template-based and *ab initio* oligomer predictions using GalaxyHomomer [27]. Outputs of the template-based (structure-based) oligomer modelling and of the *ab initio* docking results are reported, respectively, as Model No. 1 and 2, and Models No. 3, 4 and 5, along with the prediction confidence scores (TM-scores for template-based modelling; docking scores for the *ab initio* docking), the interface and buried areas calculations and the predicted dimer assemblies (in green: the experimental *h*L3HYPDH native dimer; in dark grey: the predicted dimer assemblies, structurally aligned to the native *h*L3HYPDH). The similarity between the predicted and the experimental dimeric assemblies were calculated by measuring the RMSD between the Cα of the atomic coordinates after optimal rigid body superposition. Model No. 5 reports an incorrect *ab initio* dimeric assembly prediction, consistent with the lowest docking score and unfavourable RMSD.

	*h*L3HYPDH	*tc*ProR (PDB: 1 W61)	*tl*T3LHypD (PDB: 6R76)
Interface area (Å^2^)	1914.3	1471.4	2640.1
Buried area (Å^2^)	3828.5	2942.8	5280.2
Buried area (%)	12.76	9.99	16.20

**Template-based oligomer modelling**						
**Model No.**	**Oligomer template**	**Interface area (Å^2^)**	**Buried area upon complex formation (Å^2^)**	**Structural similarity (TM-score)**	**RMSD (Å)**	**Predicted dimer assembly**
1	6R77	2124.8	3993.6	0.9255	2.4	
2	6J7C	2050.3	4100.6	0.8902	2.8	
***Ab initio* docking**						

**Model No.**	**Number of subunits**	**Interface area (Å^2^)**	**Buried area upon complex formation (Å^2^)**	**Docking score**	**RMSD (Å)**	**Predicted dimer assembly**

3	2-mer	2068.2	4136.4	1769.3	2.5	
4	2-mer	1577.7	3155.4	1321.0	6.8	
5	2-mer	1696.7	3393.4	1047.4	25.1	

In general, the oligomeric state of a protein is determined by experimental data analysis and/or by literature survey. Due to the absence of a quaternary structure in the predicted *h*L3HYPDH model and assuming any previous information concerning its native oligomeric state, we wondered whether computational tools alone might have helped the prediction of the native *h*L3HYPDH oligomerization state. For this, we used GalaxyHomomer [27], a program also used in CASP14 and part of the GalaxyWEB web server [28] that performs automated template-based modelling and *ab initio* docking for protein oligomerization prediction based on sequences coevolution criteria and conformational space annealing [29]. Template-based prediction using GalaxyHomomer performed on the predicted monomeric *h*L3HYPDH structure led to the generation of two dimeric models using the structures of *tl*T3LHypDH (PDB code: 6R77; 41.8 % identity) and of a proline racemase-like protein from *T. litoralis* (PDB code: 6J7C; 33.9 % identity) as templates. The template-based prediction correctly produced the experimentally observed dimer (Models No. 1 and No. 2 of Table 2) with a calculated interface of 2124.8 Å^2^ and 2050.3 Å^2^ for each template, corresponding to 3993.6 Å^2^ and 4100.6 Å^2^ of buried area, respectively, in close agreement with the calculated interface and buried area of the experimental *h*L3HYPDH structure (1914,2 Å^2^ and 3828.5 Å^2^) and with favourable RMSD values (2.4 Å and 2.8 Å for Model No. 1 and Model No. 2, respectively).

Consistently with the template-based prediction, the *ab initio* approach likewise produced dimeric models, with Models No. 3 and No. 4 having the highest docking scores and with favourable RMSD values (2.5 Å and 6.8 Å respectively; Table 2). The Model No.5 however reported the lowest docking score and the highest RMSD value (25.1 Å), consistent with an implausible dimeric assembly and dimer interface (Table 2 and Supplementary Table 1).

Further, we reasoned whether the native *h*L3HYPDH dimer could be correctly built using the *h*L3HYPDH AF structure as the search model in molecular replacement (MR), a computational method largely used in macromolecular crystallography for phase calculation, that aims at correctly positioning and orienting the (homologous) protein models in the unit cell [31]. We speculated whether the rotation and the translation of the monomeric computational model in the unit cell could recapitulate the native, experimental dimer, thus automatically assigning the correct symmetry and stoichiometry to the final protein model. For this, MR was performed using the monomeric AF model assigning two molecules per asymmetric unit, as suggested by the Matthews coefficient calculation. The MR performed with the program PHASER produced two solutions, one with a translation function Z-score (TFZ) of 8.5 and the number of packing clashes (PAK) of 4, and the other with a more favourable TFZ of 11.3 and a PAK value of 1. Direct refinement using the model of the second solution produced decreasing error factors, and inspection of the output model showed the monomers matching the crystallographic dimeric structure (RMSD = 2,57 Å). Automatic model building performed using AUTOBUILD [32] from PHENIX [33] covered 95 % of the final model with favourable refinement statistics (R/R_free_ = 0.24/0.28), eventually settling to R/R_free_ values of 0.22/0.25 after manual model building and refinement of the complete structure.

## 3 Discussion

The latest development of AF [5] and its remarkable accuracy in predicting protein structures [2] caused a surge in excitement about the potential and future implications of such ground-breaking milestone in the field of computational protein structure prediction. Although the number of deposited structures in the PDB is steadily increasing [34], the structural characterisation of the entire human proteome is still a long way off. Worldwide structural genomics efforts helped to accelerate the structural elucidation of the human proteome; however, AF demonstrated that the experimental approach for solving protein structures could be, at least up to a certain extent, reliably substituted by neuronal networks and artificial intelligence algorithms.

Following the release of the AF Protein Structure Database [6], here we have shown the integration of the AF model of *h*L3HYPDH with its first crystal structure, highlighting their conformational differences and the unprecedented role of a disulphide bond involving a ligand-binding cysteine, that we demonstrated having a catalytic regulatory role. Overall, the computational and experimental structures presented here provide snapshots of the transition from the ligand-free to the substrate-bound states, as already observed in experimental structures of ortholog proteins [22].

It has been previously observed that AF favours the prediction of the ligand-bound rather than the ligand-free protein conformations [35]. In general, AF predicts protein structures by performing multiple sequence alignments and coevolutionary analysis aimed at iteratively examining the evolutionary trajectories and the relative distances of the residues that are progressively interacting during structure prediction iterations. Importantly, AF does not perform energy minimizations calculations, but rather relies on a training set for deriving the structural and coevolutionary instances that associate a structure to a given sequence. This considering, it is logical to ascribe the general bias of AF for the ligand-bound conformations to having trained the algorithm on the Protein Data Bank (PDB), a database in which the number of protein structures solved by crystallographic methods largely outnumbers those solved by other techniques. Hence, AF is trained in predicting the protein structures as they would have been crystallized and as they would appear in the PDB, irrespective of energy minimization criteria. Given that the vast majority of the protein structures deposited in the PDB derive from crystallization experiments (a process that is generally favoured by the presence of protein stabilizing factors such as ligands or cofactors), it is conceivable the preference of AF for those conformations that best represent the ligand-stabilized (and more prone to crystallize) structures. Hence, AF preferentially arranges the binding site conformations as if the ligand(s) was present in the model [5], thus rationalizing, in case of the predicted *h*L3HYPDH structure, the preference of AF for the closed conformation, even in absence of the substrate.

The faithful structural arrangement of the residues of the catalytic site in the predicted *h*L3HYPDH closed structure compared to the corresponding residues of the ligand-bound homolog *tl*T3LHypD reflects the general ability of AF to reliably predict the ligand-bound arrangements of the side chains of the interacting residues, even in absence of substrate or ligands [5]. However, the comparison of the experimental open with the predicted closed structures of *h*L3HYPDH suggested a sequestering mechanism of the ligand binding Cys104 via an intramolecular disulphide bond formation with Cys137, leading to speculations on the catalytic and regulatory role of Cys104 as well as the reversibility of the disulphide bond under favourable conditions. Previous experiments already showed that *h*L3HYPDH is active in absence of reducing agents [15], a condition that, as observed in the experimental *h*L3HYPDH structure, promotes the formation of the disulphide bond by making unavailable the Cys104 for ligand binding. Conversely, the reducing environment favours the reduction of the disulphide bond and the flipping of Cys104 toward the catalytic centre, as suggested by the computational model. Thus, assuming the closed state as the only active conformation of *h*L3HYPDH irrespective of the redox environment, the oxidizing condition favours the widening of the catalytic pocket by positioning the Cys104 away from the catalytic site and promoting the formation of the disulphide bond, thus decreasing the enzyme interacting capacity with the substrate and enhancing its catalytic rate. On the contrary, the reducing condition reverses this situation by favouring the flipping of Cys104 toward the catalytic site, thus increasing the enzyme interacting capacity with the substrate. Since the *K*_M_ can be loosely interpreted as a descriptor of the affinity between an enzyme and its substrate, such redox-dependent interacting capacity between the enzyme and the substrate is reflected by the shifting of the *K*_M_ to higher values (i.e. lower affinity) when measured under oxidizing conditions, and to lower values (i.e. higher affinity) when under reducing conditions. This enzyme behaviour is also mirrored by the *k*_cat_/*K*_M_ ratios, which indicate a higher catalytic efficiency of the enzyme under reducing conditions compared to the oxidizing conditions. Moreover, our data shows that substrate inhibition is only observed under reducing conditions, further evidencing the increased interacting capacity of the enzyme with the substrate due to the flipping of the substrate-interacting Cys104.

The experimentally determined *h*L3HYPDH structure allowed the identification of a specific structural element (i.e. the Cys104-Cys137 disulphide bond) that was absent in the predicted model and that our data demonstrated its functional role, thus highlighting the complementarity of the experimental and computational protein solution and prediction for protein functional and structural analysis. Hence, by combining the experimental and the AF structures of *h*L3HYPDH and interpreting them in light of the catalytic data, meaningful assumptions could be inferred regarding the enzyme catalysis and regulation.

One of the applications of the AF models is their use in MR for structure solution of structurally unknown proteins or for proteins for which the molecular replacement is hampered by the poor homology or the inadequacy of the search model. We used the unmodified, monomeric AF model for MR, structure solution and model building, leading to the automatic completion of nearly 95 % of the dimeric enzyme. Hence, our case shows that the computational *h*L3HYPDH AF structure streamlined the at times laborious selection and/or modification of the search model for MR, resulting in the correct arrangement of the MR output model in the native dimeric form, an information that was missing in the predicted structure and that publicly available servers providing template-based and *ab initio* computational methods for oligomer prediction were able to recover, as reported above.

While AF offers to the scientific community the most reliable algorithm to date for predicting protein structures, the predicted models are generally biased towards those conformations that are more prone to crystallization, a direct consequence of having selected the PDB as the training set, a database where the crystal structures account for more than 87 % of the total deposited coordinates [36]. However, this could come at hand (also retrospectively) for rescuing and reprocessing those crystallographic data that failed during MR and for which the AF structures could constitute valid search models [37].

In perspective, it can be envisaged that the increasing number of Cryo-EM structures deposited in the PDB could skew the current bias of AF toward less crystal-oriented structures to a more conformationally varied models. Regardless, feeding the AF models in automatic structure solution pipelines could significantly enhance structural and functional analysis of structurally unsolved proteins, thus advancing the developing field of integrative structural biology.

## 4 Methods

*Protein Expression and Purification*. The human *trans*-3-hydroxy-l-proline dehydratase gene (Uniprot ID: Q96EM0) was cloned in pET28b vector and expressed in *E. coli* BL21(DE3) cells. Bacteria were grown on agar plate, precultured overnight and then diluted in 1 L of 2xTY medium. The optical density was constantly monitored until it reached 0.6 when the temperature was then shifted to 20 °C, and protein expression was induced overnight by the addition of 0.5 mM isopropyl 1-thio-β-d-galactopyranoside. The cells were then pelleted and resuspended in 30 ml of 1xPBS buffer at pH 7.4 and lysed following 8 cycles of sonication. Pellet and supernatant were separated by centrifugation, and the supernatant was applied to a preequilibrated His-Trap^TM^ column (Cytiva) and eluted with a linear gradient of imidazole. The protein was then loaded on a Superdex 200 Increase 10/300 GL equilibrated with 50 mM Tris pH = 8, 50 mM NaCl for the final purification step. The purified protein solution was aliquoted and frozen at −80 °C until further use.

*Protein Crystallization and Structure Solution.* For initial crystal screening, purified *h*L3HYPDH was concentrated to 14 mg/ml using Vivaspin concentrators (Sartorius AG) with a molecular mass cut-off of 50 kDa. Crystallization screens were performed using an Oryx4 Protein Crystallization Robot (Douglas Instruments ltd.) and the Classics Suite I (Qiagen AG) and the Structure Screen and the Morpheus Screen (Molecular Dimensions U.K. ltd.), with and without the substrate or the proline racemase inhibitor pyrrole-2-carboxylic acid (PYC) [23], both at 1 mM concentration. Initial crystals grew in a solution containing 0.1 M MES pH = 6.5 and 12 % (w/v) PEG 20000, and manual crystal optimisation was performed varying the pH (6.1–6.7), the concentration of PEG 20,000 (6 %-20 %) and the protein concentration (8 mg/ml and 14 mg/ml). Optimized crystals grew after one-month incubation at 20 °C temperature and were cryoprotected with 12 % glycerol and flash frozen in liquid nitrogen for diffraction experiments. Best crystals diffracted at 3.0 Å resolution at beamline ID30B at the Electro Synchrotron Research Facility (ESRF; Grenoble) [38]. Data were processed using XDS [39] and scaled using SCALA [40], and automated search model generation and molecular replacement (MR) were automatically performed using, respectively, MrBUMP [41] and PHASER [42] of the CCP4 web application [43], identifying the structure of *T. litoralis trans*-3-Hydroxy-l-proline dehydratase as the best search model (Protein Data Bank ID code: 6R77). For MR, the *h*L3HYPDH AF structure [21] was also used as the search model, as described in the paper. Automatic model building was performed using AUTOBUILD [32] of the PHENIX [33] suite. The final structure was manually built using COOT [44], refined by REFMAC [45], and validated using MOLPROBITY [46]. All molecular graphics images were produced using PyMOL [47]. Structure and sequence alignments were performed using Clustal Omega [25] and edited with ESPript [26].

*Enzyme activity assay. h*L3HYPDH activity was measured using a coupled-enzyme assay using hydroxyproline as the substrate and the NAD-dependent *T. litoralis* Pyr2C reductase (*tl*Pyr2C) [14] as the secondary enzyme. The standard assay solution contained 10 μg of *h*L3HYPDH and 10 μg of *tl*Pyr2C diluted in 1xPBS in 200 μl final volume, and reducing conditions were produced by adding 1 mM of dithiothreitol (DTT) to the reaction mixture. The addition of DTT had no effect on *tl*Pyr2C activity (data not shown). NADH oxidation was monitored at 340 nm wavelength using a TECAN Sunrise Microplate Reader (Tecan Trading AG, Switzerland). Since the Michaelis-Menten curve measured in reducing condition showed substrate inhibition at the highest substrate concentration, all points were interpolated using the substrate inhibition kinetics of GraphPad Prism [48].

*Bioinformatic analysis.* Computational protein oligomerization predictions were performed using GalaxyHomomer [27] of the GalaxyWEB platform [28], and the buried area interfaces were measured using the COCOMAPS server [30]. Distance-difference matrix was produced using PHENIX [33]. Protein structure and interaction network analysis were performed both manually and using the ProteinTools server [49].

*Protein sulfhydryls blocking.* N-ethylmaleimide (NEM) at a final concentration of 20 mM was dissolved in 50 mM phosphate buffer containing 6 M guanidinium chloride, reaching a final pH of 7.3. For the blocking reaction of the free cysteines, an equivalent volume of the solution containing NEM and urea was added to the protein solution and incubated for 10 min at room temperature. The reaction was quenched adding trifluoroacetic acid to a final concentration of 0.3 %.

*Mass spectrometry analysis.* Protein masses were determined by LCMS using an Aquity UPLC system (Waters) linked to a Q-Exactive Plus mass spectrometer. A BioResolve RP mAB Polyphenyl Column (2.1x50 mm) was developed with a gradient comprising 0.1 % formic acid (FA) (Buffer A) and 0.5 % FA in acetonitrile (Buffer B) at a flow of 0.4 ml/min and using the following gradient: 5 % Buffer B, 0.5 min; 15 % Buffer B, 9 min; 60 % Buffer B, 10 min; 80 % Buffer B, 11 min; 5 % Buffer B. The mass spectrometer was operated in positive mode with resolution set to 280,000 and *m*/*z* range from 800 to 6000. Automatic Gain Control (AGC) and maximum injection time were set to 3x10^6^ and 200 msec, respectively. Raw data were processed with BioPharma Finder Software (Thermo Fisher), using the Xtract option with sliding window.

*PDB Deposition.* The coordinates and the structure factors were deposited in the Protein Data Bank under ID code 7QPO.

## CRediT authorship contribution statement

**Eugenio Ferrario:** Investigation, Formal analysis, Data curation, Validation, Writing - review & editing. **Riccardo Miggiano:** Validation, Investigation, Formal analysis, Data curation, Writing - review & editing. **Menico Rizzi:** Funding acquisition, Supervision, Writing - review & editing. **Davide M. Ferraris:** Conceptualization, Validation, Investigation, Formal analysis, Visualization, Data curation, Project administration, Methodology, Resources, Supervision, Writing - original draft, Writing - review & editing.

## Declaration of Competing Interest

The authors declare that they have no known competing financial interests or personal relationships that could have appeared to influence the work reported in this paper.
