# Supplementary material for: The integration of AlphaFold-predicted and crystal structures of human trans-3-hydroxy-l-proline dehydratase reveals a regulatory catalytic mechanism
Source: Comput Struct Biotechnol J. 2022 Jul 18;20:3874–83. doi: 10.1016/j.csbj.2022.07.027 (PMC9309405; doi:10.1016/j.csbj.2022.07.027)
Supplement: Supplementary data 1 [file mmc1.docx]

**Supplementary Material**

**The integration of AlphaFold-predicted and crystal structures of human *trans*-3-Hydroxy-L-proline dehydratase reveals a regulatory catalytic mechanism**

Eugenio Ferrario^1,2^, Riccardo Miggiano^1,3^, Menico Rizzi^1^, Davide M. Ferraris^1,3*^

^1^Università del Piemonte Orientale, Dipartimento di Scienze del Farmaco, Novara, Italy

^2^Present address: Department of Biomedicine, University of Bergen, Bergen, 5009, Norway.

^3^IXTAL srl, Via Bovio 6, 28100, Novara, Italy

The Supplementary Material contains Supplementary Figures 1-6, Supplementary Table 1 and captions of Supplementary Video 1, Supplementary Video 2, Supplementary Video 3 and Supplementary Video 4.


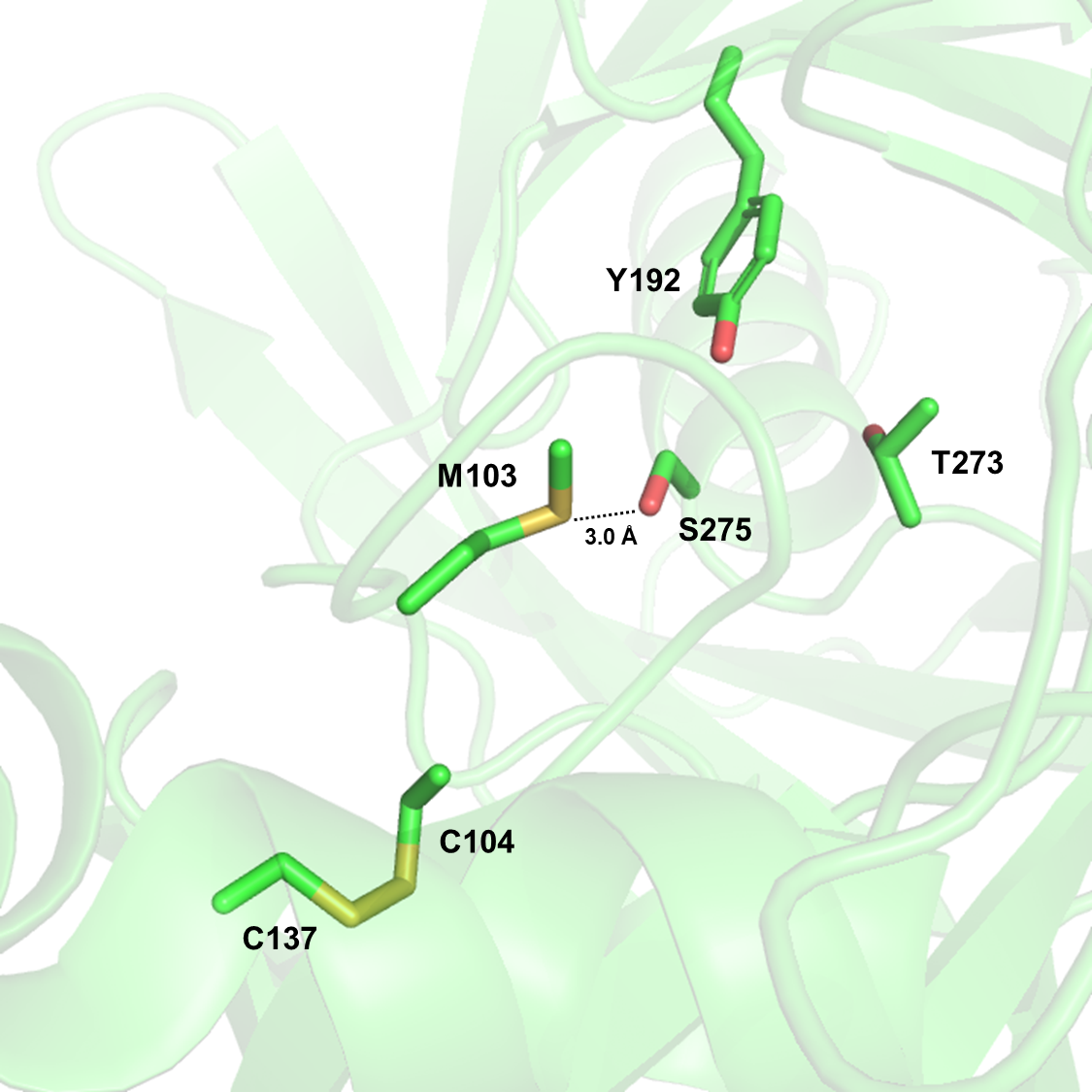


**Supplementary Figure 1.** Spatial arrangement of the ligand binding residues in the experimental open conformation, showing the disulphide bridge between C104 and C137 and the stabilizing interaction between M103 and S275.


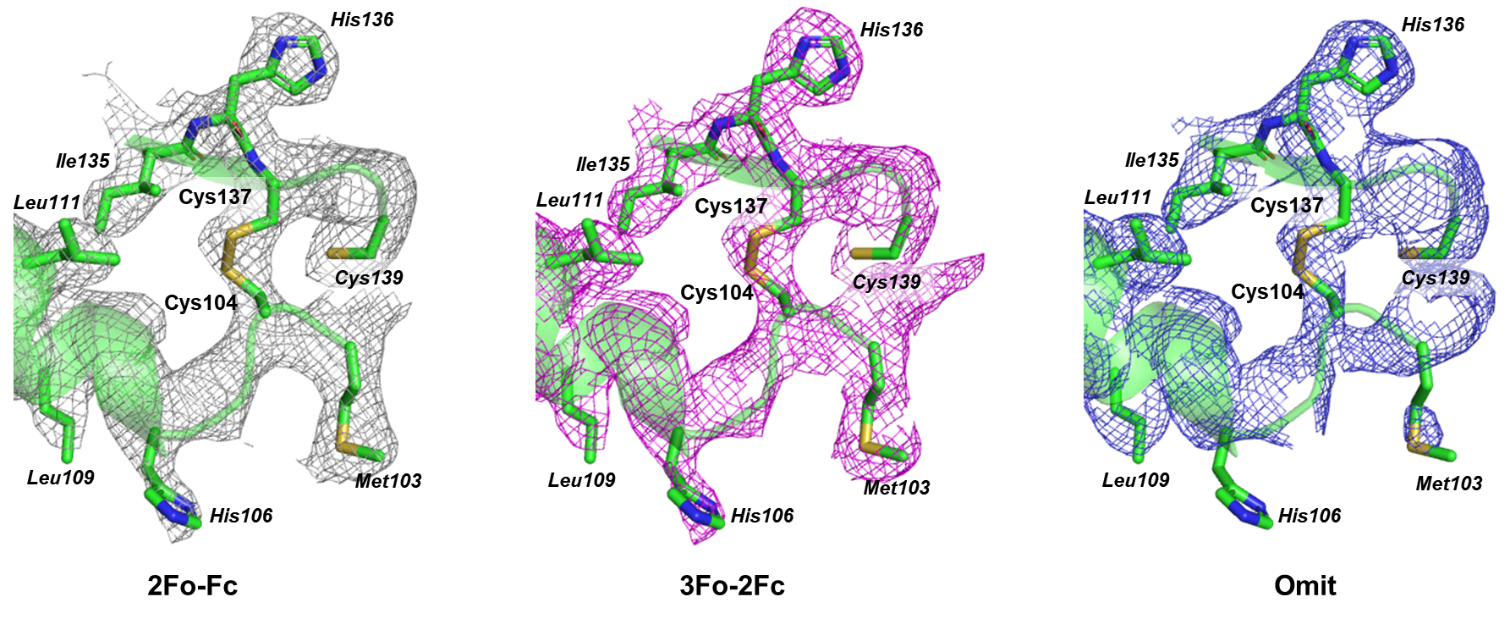


**Supplementary Figure 2.** From left to right, 2Fo-Fc map (contoured at 1.0 σ), 3Fo-2Fc map (2.0 σ) and omit map (2.0 σ). In bold, Cys104 and Cys137 involved in the intramolecular disulphide bond; in italics, representative amino acids and related electron density, showing that Cys139 does not engage in disulphide bond formation.


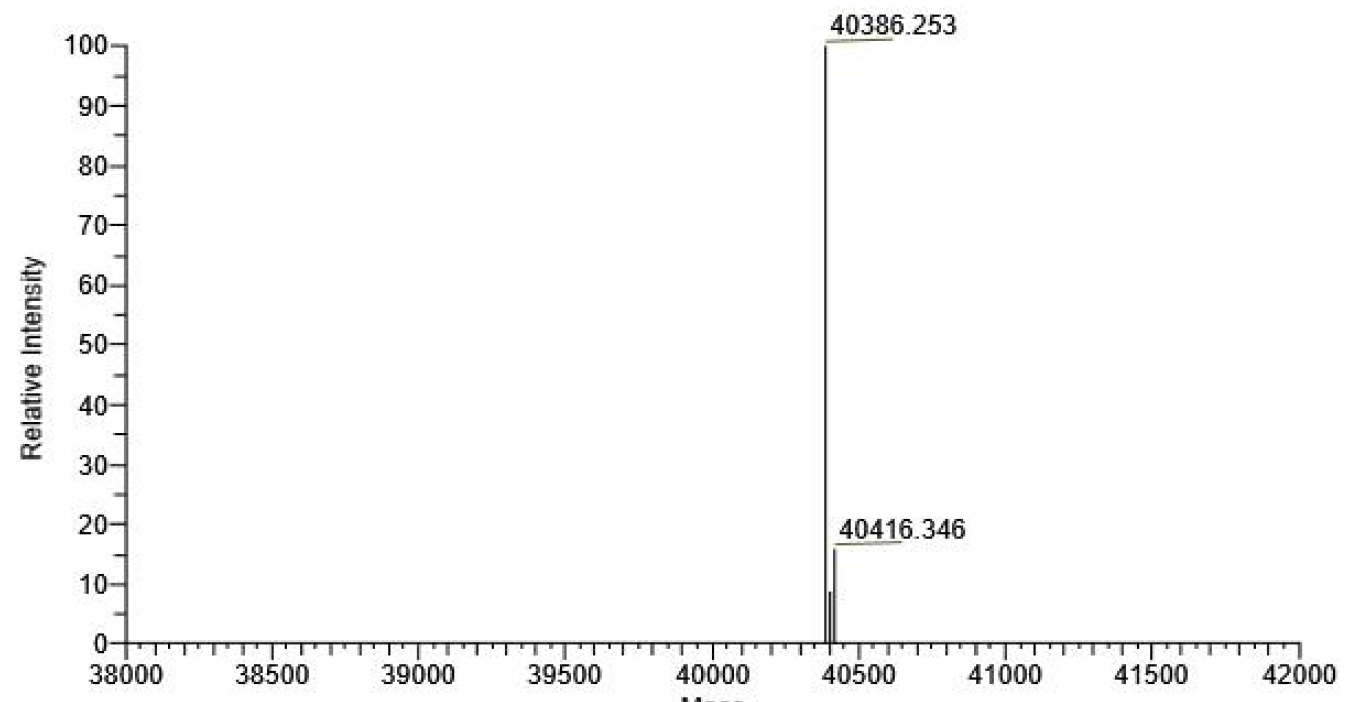


| **Monoisotopic Mass** | **Relative Abundance** |
| --- | --- |
| 40386.958 | 100.0% |
| 40416.346 | 16.2% |

**Supplementary Figure 3.** Deconvoluted mass spectrum of the NEM-untreated, non-reduced *h*L3HYPDH sample and table reporting the masses and relative abundances. The discrepancy of this experimental mass with the theoretical mass of *h*L3HYPDH (40543.25 Da) is likely due to unspecific N-terminal methionine excision.

**
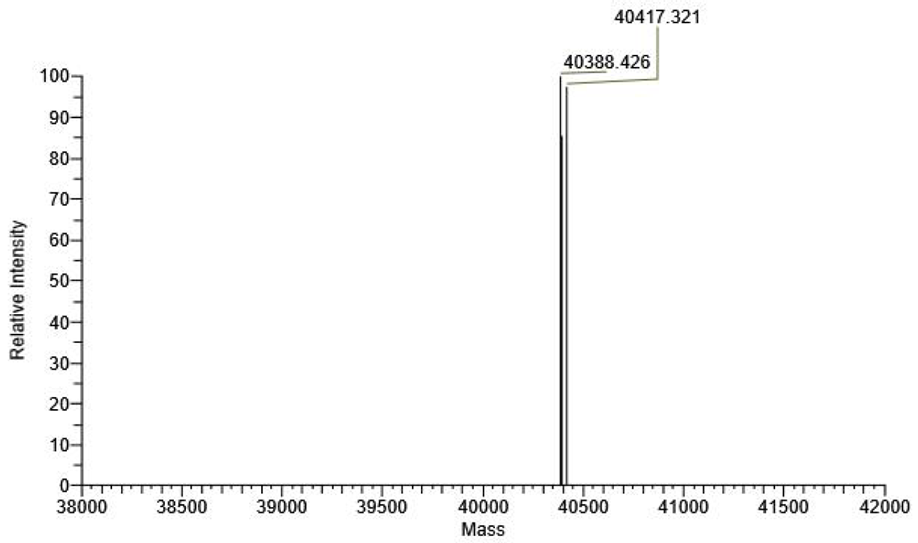
**

| **Monoisotopic Mass** | **Relative Abundance** |
| --- | --- |
| 40388.426 | 100.0% |
| 40417.321 | 97.64% |

**Supplementary Figure 4.** Deconvoluted mass spectrum of the NEM-untreated, reduced *h*L3HYPDH sample and table reporting the masses and relative abundances.


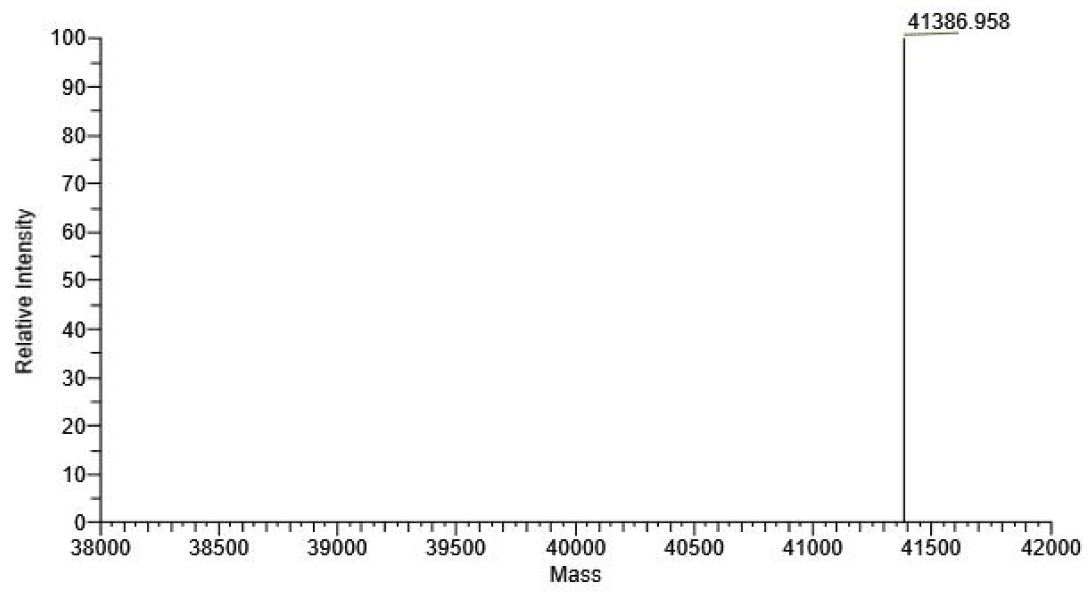


| **Monoisotopic Mass** | **Relative Abundance** | **Mass difference with the untreated sample** | **No. of NEM incorporated** |
| --- | --- | --- | --- |
| 41386.958 | 100.0% | 998,532 | 8 |

**Supplementary Figure 5.** Deconvoluted mass spectrum of the reduced, NEM-treated *h*L3HYPDH sample, and table reporting the masses and relative abundances.

Mass spectrometry analysis of the non-reduced *h*L3HYPDH treated with NEM (cfr. Materials and Methods) produced species with different masses, the most abundant having masses of 41260.78 (abundance: 100%), 41135,81 (abundance: 91,36%), compatible with the incorporation of 6/7 NEM (125 Da) into free cysteines, suggesting the presence of one disulfide-bridge in *h*L3HYPDH, theoretically having 8 NEM molecules added to the fully reduced *h*L3HYPDH, while the non-reduced protein would have 6 added cysteines.


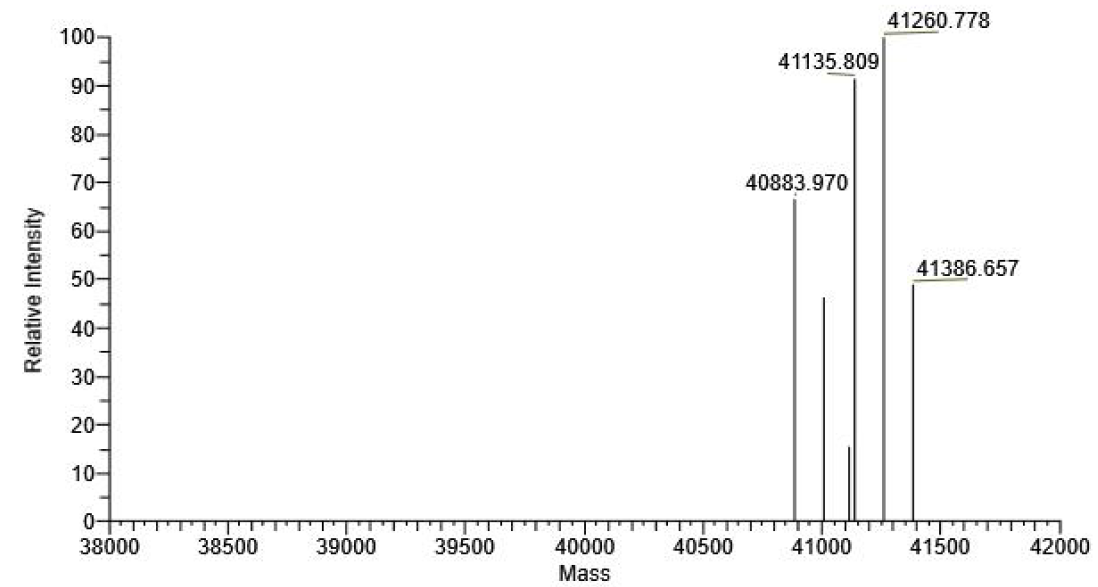


| **Monoisotopic Mass** | **Relative Abundance** | **Mass difference with the untreated sample** | **No. of NEM incorporated** |
| --- | --- | --- | --- |
| 41260.778 | 100.0% | 874.525 | 7 |
| 41135.809 | 91.4% | 749.556 | 6 |

**Supplementary Figure 6.** Deconvoluted mass spectrum of the non-reduced *h*L3HYPDH treated with NEM and table reporting the masses and relative abundances.

**Captions of Supplementary Videos**

**Supplementary Video 1.** Conformational dynamics of the open and closed states of *h*L3HYPDH, obtained by morphing the experimental open monomer and the AlphaFold close structure.

**Supplementary Video 2.** Side view of the conformational morphing of Supplementary Video 1.

**Supplementary Video 3.** Dynamics of residues stabilizing the open and the closed conformations of *h*L3HYPDH. The dashed line indicates the interatomic distances separating the interacting residues E98 and Q267 in the open conformation and the residues Y76 and Y241 in the closed conformation.

**Supplementary Video 4.** Dynamics of the catalytic site residues during the transition from the open to the closed conformation, as derived from the experimental and predicted *h*L3HYPDH structures.
