# Supplementary material for: The integration of AlphaFold-predicted and crystal structures of human trans-3-hydroxy-l-proline dehydratase reveals a regulatory catalytic mechanism
Source: Comput Struct Biotechnol J. 2022 Jul 18;20:3874–83. doi: 10.1016/j.csbj.2022.07.027 (PMC9309405; doi:10.1016/j.csbj.2022.07.027)
Supplement: Supplementary data 6 [file mmc6.pdf]

hL3HYPDH crystal structure

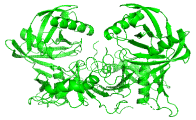

PRO	11	CB	A	TYR	333	CD1	B	3.29
PRO	12	CG	A	ASP	117	OD2	B	3.11
HIS	26	NE2	A	ASP	345	OD2	B	2.97
GLY	29	O	A	PHE	351	CZ	B	3.49
GLU	68	OE2	A	ARG	270	NE	B	3.41
ARG	70	NE	A	THR	334	O	B	3.26
ARG	113	NH2	A	GLU	342	OE2	B	2.59
ASP	117	O	A	ARG	9	NH1	B	2.92
SER	206	O	A	LYS	354	NZ	B	2.83
THR	209	OG1	A	LYS	354	O	B	2.68
ARG	270	NE	A	GLU	68	OE1	B	2.63
ARG	270	NH1	A	LEU	353	CD2	B	3.15
THR	303	CG2	A	PHE	351	CE1	B	3.41
HIS	332	ND1	A	GLU	342	OE1	B	3.05
TYR	333	N	A	GLU	342	OE1	B	3.08
THR	334	O	A	ILE	340	N	B	3.14
THR	334	O	A	ARG	70	NE	B	3.20
GLY	335	CA	A	SER	338	O	B	3.08
THR	336	O	A	SER	338	N	B	2.69
THR	336	O	A	ALA	337	CA	B	3.07
THR	336	OG1	A	PRO	11	CD	B	3.44
ALA	337	CA	A	THR	336	O	B	3.08
SER	338	N	A	THR	336	O	B	2.77
SER	338	O	A	GLY	335	CA	B	3.13
ILE	340	N	A	THR	334	O	B	3.08
GLU	342	OE1	A	HIS	332	ND1	B	2.84
GLU	342	OE2	A	ARG	113	NH2	B	2.96
GLU	342	OE1	A	TYR	333	N	B	3.37
ASP	345	OD2	A	HIS	26	NE2	B	2.94
LEU	347	CD1	A	THR	303	CG2	B	3.28
PHE	351	CE1	A	THR	303	OG1	B	3.44
LYS	354	O	A	THR	209	OG1	B	3.47

Model 1 RMSD=2.392 (595 to 595 atoms)

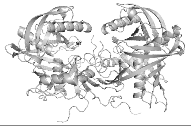

LEU	10	CD2	A	THR	336	OG1	B	3.28
HIS	26	NE2	A	PRO	69	O	B	2.66
GLY	29	H	A	LEU	347	CD1	B	3.29
GLY	29	O	A	PHE	351	CZ	B	3.42
GLU	68	OE2	A	ARG	270	HH2	B	1.89
PRO	69	O	A	THR	334	HG1	B	2.42
PRO	69	O	A	HIS	26	NE2	B	2.67
ARG	70	HE	A	THR	334	O	B	3.04
ARG	73	HH1	A	ARG	210	HH2	B	3.12
ARG	113	HH2	A	GLU	342	OE1	B	1.90
LYS	208	HZ1	A	ASP	214	OD2	B	2.46
THR	209	H	A	LYS	354	OXT	B	2.10
ARG	210	HH1	A	ASP	214	OD1	B	2.10
ARG	210	HH1	A	GLU	266	OE2	B	2.46
ARG	210	HH1	A	VAL	213	CG1	B	2.75
ARG	210	HH2	A	ARG	73	HH1	B	3.12
ARG	210	HH1	A	ARG	210	O	B	3.38
VAL	213	CG1	A	ARG	210	HH1	B	2.76
ASP	214	OD1	A	ARG	210	HH1	B	2.10
ASP	214	OD2	A	LYS	208	HZ1	B	2.47
GLU	266	OE2	A	ARG	210	HH1	B	2.45
ARG	270	HH2	A	GLU	68	OE2	B	1.88
ARG	270	HE	A	LEU	353	CD1	B	3.13
ARG	270	CD	A	PHE	351	CE1	B	3.48
HIS	332	ND1	A	GLU	342	OE2	B	2.85
HIS	332	NE2	A	PRO	346	CD	B	3.39
TYR	333	H	A	GLU	342	OE1	B	1.96
THR	334	O	A	ILE	340	H	B	2.01
THR	334	HG1	A	PRO	69	O	B	2.41
THR	334	O	A	ARG	70	HE	B	3.03
THR	334	O	A	PHE	339	CD2	B	3.27
GLY	335	CA	A	ILE	340	H	B	3.22
THR	336	O	A	SER	338	H	B	1.84
THR	336	OG1	A	LEU	10	CD2	B	3.28
SER	338	H	A	THR	336	O	B	1.84
PHE	339	CD2	A	THR	334	O	B	3.27
ILE	340	H	A	THR	334	O	B	2.01
ILE	340	H	A	GLY	335	CA	B	3.23
GLU	342	OE1	A	ARG	113	HH2	B	1.92
GLU	342	OE1	A	TYR	333	H	B	1.97
GLU	342	OE2	A	HIS	332	ND1	B	2.85
PRO	346	CD	A	HIS	332	NE2	B	3.42
LEU	347	CD1	A	GLY	29	H	B	3.31
PHE	351	CZ	A	GLY	29	O	B	3.44
PHE	351	CE1	A	ARG	270	CD	B	3.49
LEU	353	CD1	A	ARG	270	HE	B	3.13
LYS	354	OXT	A	THR	209	H	B	2.11

Model 2 RMSD=2.767 (599 to 599 atoms)

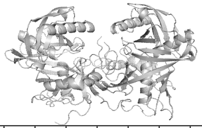

ALA	4	CB	A	GLY	119	O	B	3.47
LEU	5	CD2	A	LEU	120	CD2	B	3.08
ARG	9	O	A	PHE	118	CZ	B	3.27
PRO	11	CB	A	TYR	333	CD2	B	2.44
PRO	12	CA	A	ARG	152	NH2	B	1.51
PRO	12	CD	A	ASP	117	OD2	B	3.20
HIS	13	N	A	ARG	152	NH2	B	2.28
HIS	26	CG	A	PRO	69	CG	B	2.83
GLU	68	OE2	A	ARG	270	NE	B	2.95
PRO	69	CG	A	HIS	26	CG	B	2.83
ARG	70	CG	A	THR	334	CB	B	2.98
ARG	70	NH2	A	TYR	333	O	B	3.37
ASP	117	OD2	A	PRO	12	CD	B	3.20
PHE	118	CZ	A	ARG	9	O	B	3.27
GLY	119	O	A	ALA	4	CB	B	3.47
LEU	120	CD2	A	LEU	5	CD2	B	3.08
ARG	152	NH2	A	PRO	12	CA	B	1.51
ARG	152	NH2	A	HIS	13	N	B	2.28
ARG	270	NE	A	GLU	68	OE2	B	2.95
ALA	302	O	A	LEU	353	CD2	B	1.90
THR	303	CG2	A	PHE	351	CE2	B	3.10
GLN	330	NE2	A	ASP	344	OD2	B	2.43
HIS	332	CE1	A	ASP	345	CA	B	2.68
TYR	333	CD2	A	PRO	11	CB	B	2.44
TYR	333	CB	A	ILE	340	CD1	B	2.58
TYR	333	CD2	A	GLU	342	OE1	B	2.90
TYR	333	O	A	ARG	70	NH2	B	3.37
THR	334	O	A	PHE	339	CD1	B	2.13
THR	334	CB	A	ARG	70	CG	B	2.98
THR	334	O	A	ILE	340	N	B	3.31
GLY	335	CA	A	SER	338	O	B	1.90
GLY	335	CA	A	PHE	339	CA	B	3.19
THR	336	O	A	ALA	337	CA	B	1.15
THR	336	O	A	SER	338	N	B	2.00
THR	336	O	A	THR	336	O	B	3.12
ALA	337	CA	A	THR	336	O	B	1.16
ALA	337	CA	A	ALA	337	CA	B	2.85
SER	338	O	A	GLY	335	CA	B	1.90
SER	338	N	A	THR	336	O	B	2.00
PHE	339	CD1	A	THR	334	O	B	2.13
PHE	339	CA	A	GLY	335	CA	B	3.19
ILE	340	CD1	A	TYR	333	CB	B	2.58
ILE	340	N	A	THR	334	O	B	3.31
GLU	342	OE1	A	TYR	333	CD2	B	2.90
ASP	344	OD2	A	GLN	330	NE2	B	2.43
ASP	345	CA	A	HIS	332	CE1	B	2.68
PHE	351	CE2	A	THR	303	CG2	B	3.10
LEU	353	CD2	A	ALA	302	O	B	1.90

Model 3 RMSD=2.530 (617 to 617 atoms)

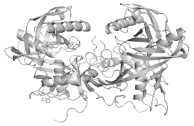

LEU	10	CD2	A	THR	336	OG1	B	3.30
PRO	11	CB	A	TYR	333	CD1	B	2.74
PRO	11	CG	A	VAL	23	CG1	B	3.31
PRO	11	CG	A	PHE	118	CZ	B	3.48
PRO	12	CB	A	ARG	152	NH2	B	2.65
PRO	12	CD	A	TYR	333	CE2	B	3.00
PRO	12	CG	A	ASP	117	OD2	B	3.16
HIS	13	N	A	ARG	152	NH2	B	3.31
VAL	23	CG1	A	PRO	11	CG	B	3.29
HIS	26	NE2	A	ASP	345	OD1	B	1.73
HIS	26	CE1	A	LEU	347	CB	B	2.86
HIS	26	CB	A	PRO	69	CG	B	3.19
GLY	28	C	A	LEU	347	CD1	B	2.97
GLY	29	CA	A	LEU	347	CD1	B	2.11
GLY	29	O	A	PHE	351	CE1	B	2.81
GLU	68	OE2	A	ARG	270	NE	B	2.30
PRO	69	CG	A	HIS	26	CB	B	3.19
ARG	70	NH2	A	THR	334	CG2	B	2.57
ARG	70	NH2	A	TYR	333	O	B	3.21
GLY	71	O	A	HIS	72	CD2	B	2.65
HIS	72	CB	A	HIS	72	CB	B	2.52
HIS	72	CD2	A	GLY	71	O	B	2.65
ARG	113	NH2	A	ASP	344	OD2	B	3.36
ASP	117	OD2	A	PRO	12	CG	B	3.15
PHE	118	CZ	A	PRO	11	CG	B	3.50
ARG	152	NH2	A	PRO	12	CB	B	2.67
ARG	152	NH2	A	HIS	13	N	B	3.32
THR	209	OG1	A	LYS	354	O	B	2.49
ARG	270	NE	A	GLU	68	OE2	B	2.30
ARG	270	CZ	A	LEU	353	CD1	B	2.41
ARG	270	CG	A	PHE	351	CE2	B	2.68
ALA	302	CB	A	LEU	353	CD2	B	3.33
GLN	330	NE2	A	ASP	344	OD2	B	1.87
ALA	331	O	A	GLU	342	OE2	B	3.25
HIS	332	CE1	A	ASP	344	C	B	1.89
HIS	332	CE1	A	ASP	345	N	B	2.09
HIS	332	NE2	A	PRO	346	CD	B	2.77
HIS	332	CA	A	GLU	342	OE2	B	2.97
TYR	333	CD2	A	GLU	342	OE1	B	1.74
TYR	333	CB	A	ILE	340	CD1	B	2.60
TYR	333	CD1	A	PRO	11	CB	B	2.71
TYR	333	CE2	A	PRO	12	CD	B	2.97
TYR	333	O	A	ARG	70	NH2	B	3.23
THR	334	O	A	ILE	340	N	B	2.28
THR	334	CG2	A	ARG	70	NH2	B	2.57
THR	334	O	A	PHE	339	C	B	2.89
GLY	335	C	A	SER	338	O	B	2.11
GLY	335	CA	A	ILE	340	N	B	2.64
GLY	335	CA	A	PHE	339	CA	B	2.77
THR	336	O	A	ALA	337	C	B	2.05
THR	336	O	A	SER	338	N	B	1.11
THR	336	OG1	A	LEU	10	CD2	B	3.28
THR	336	N	A	PHE	339	N	B	3.50
ALA	337	CB	A	ALA	337	CB	B	1.84
ALA	337	C	A	THR	336	O	B	2.06
ALA	337	N	A	SER	338	N	B	3.14
SER	338	N	A	THR	336	O	B	1.12
SER	338	O	A	GLY	335	C	B	2.12
SER	338	N	A	ALA	337	N	B	3.14
PHE	339	CA	A	GLY	335	CA	B	2.76
PHE	339	C	A	THR	334	O	B	2.91
PHE	339	N	A	THR	336	N	B	3.50
ILE	340	N	A	THR	334	O	B	2.30
ILE	340	CD1	A	TYR	333	CB	B	2.61
ILE	340	N	A	GLY	335	CA	B	2.64
GLU	342	OE1	A	TYR	333	CD2	B	1.72
GLU	342	OE2	A	HIS	332	CA	B	2.98
GLU	342	OE2	A	ALA	331	O	B	3.26
ASP	344	C	A	HIS	332	CE1	B	1.89
ASP	344	OD2	A	GLN	330	NE2	B	1.89
ASP	344	OD2	A	ARG	113	NH2	B	3.35
ASP	345	OD1	A	HIS	26	NE2	B	1.73
ASP	345	N	A	HIS	332	CE1	B	2.09
PRO	346	CD	A	HIS	332	NE2	B	2.76
LEU	347	CD1	A	GLY	29	CA	B	2.11
LEU	347	CB	A	HIS	26	CE1	B	2.85
LEU	347	CD1	A	GLY	28	C	B	2.97
PHE	351	CE2	A	ARG	270	CG	B	2.68
PHE	351	CE1	A	GLY	29	O	B	2.81
LEU	353	CD1	A	ARG	270	CZ	B	2.42
LEU	353	CD2	A	ALA	302	CB	B	3.33
LYS	354	O	A	THR	209	OG1	B	2.49

Model 4 RMSD=6.833 (616 to 616 atoms)

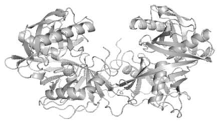

MET	1	CB	A	PRO	82	CB	B	2.62
MET	1	SD	A	SER	83	N	B	2.74
LEU	5	CD1	A	LEU	80	O	B	1.63
LEU	5	O	A	LEU	36	O	B	2.55
LEU	5	O	A	ALA	37	CA	B	2.90
LEU	5	CD2	A	VAL	35	O	B	3.18
VAL	7	C	A	ALA	37	CB	B	3.14
PRO	8	CG	A	SER	21	OG	B	2.43
PRO	8	N	A	ALA	37	CB	B	2.80
ARG	9	O	A	LEU	36	CD1	B	2.94
PRO	11	CB	A	PHE	118	CZ	B	2.60
PRO	11	CG	A	VAL	23	CG2	B	3.03
PRO	11	CD	A	THR	336	CG2	B	3.12
PRO	12	CG	A	ASP	117	O	B	3.19
SER	21	OG	A	PRO	8	CG	B	2.45
VAL	23	CG2	A	PRO	11	CG	B	3.03
VAL	35	O	A	LEU	5	CD2	B	3.20
LEU	36	O	A	LEU	5	O	B	2.57
LEU	36	CD1	A	ARG	9	O	B	2.95
ALA	37	CB	A	PRO	8	N	B	2.81
ALA	37	CA	A	LEU	5	O	B	2.92
ALA	37	CB	A	VAL	7	C	B	3.15
LEU	80	O	A	LEU	5	CD1	B	1.63
PRO	82	CB	A	MET	1	CB	B	2.63
SER	83	N	A	MET	1	SD	B	2.75
ASP	117	O	A	PRO	12	CG	B	3.18
PHE	118	CZ	A	PRO	11	CB	B	2.59
ARG	152	NH2	A	ASP	344	OD1	B	1.97
ARG	152	NH2	A	GLU	342	OE2	B	2.50
ARG	152	NH1	A	ASP	343	OD1	B	2.53
TYR	333	CD2	A	GLU	342	OE1	B	3.26
THR	336	OG1	A	SER	338	O	B	1.98
THR	336	CG2	A	PRO	11	CD	B	3.13
THR	336	O	A	ALA	337	CB	B	3.28
ALA	337	CB	A	THR	336	O	B	3.28
SER	338	O	A	THR	336	OG1	B	1.99
GLU	342	OE2	A	ARG	152	NH2	B	2.50
GLU	342	OE1	A	TYR	333	CD2	B	3.26
ASP	343	OD1	A	ARG	152	NH1	B	2.51
ASP	344	OD1	A	ARG	152	NH2	B	1.98

Model 5 RMSD=25.052 (662 to 662 atoms)

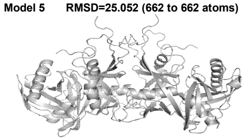

SER	3	OG	A	GLU	342	OE2	B	2.88
LEU	5	CB	A	GLU	342	OE1	B	1.48
LEU	5	CD2	A	PRO	12	CD	B	2.67
LEU	5	O	A	ILE	340	CD1	B	3.04
ALA	6	N	A	GLU	342	OE1	B	3.22
PRO	8	CG	A	SER	338	O	B	2.12
PRO	12	CD	A	LEU	5	CD2	B	2.70
PRO	82	CB	A	GLY	151	CA	B	3.00
PRO	82	CG	A	ASP	150	O	B	3.43
SER	83	O	A	PRO	124	O	B	2.55
SER	83	N	A	ALA	123	CB	B	2.96
GLU	84	O	A	ALA	126	N	B	3.05
GLU	84	O	A	PRO	125	CA	B	3.13
LEU	85	O	A	ALA	123	O	B	3.15
PRO	86	CB	A	PRO	125	CG	B	2.72
ALA	88	O	A	ALA	123	N	B	2.27
ALA	88	O	A	PRO	122	C	B	2.97
HIS	89	O	A	VAL	121	O	B	2.55
HIS	89	CD2	A	PRO	122	CG	B	2.77
HIS	89	O	A	ALA	123	N	B	3.23
PHE	118	O	A	LEU	120	CD2	B	1.94
GLY	119	O	A	LEU	120	CB	B	1.97
LEU	120	CD2	A	PHE	118	O	B	1.95
LEU	120	CB	A	GLY	119	O	B	1.97
LEU	120	CA	A	LEU	120	CA	B	2.15
LEU	120	O	A	VAL	121	N	B	3.00
VAL	121	O	A	HIS	89	O	B	2.55
VAL	121	N	A	LEU	120	O	B	3.00
PRO	122	CG	A	HIS	89	CD2	B	2.77
PRO	122	C	A	ALA	88	O	B	2.97
ALA	123	N	A	ALA	88	O	B	2.27
ALA	123	CB	A	SER	83	N	B	2.96
ALA	123	O	A	LEU	85	O	B	3.15
ALA	123	N	A	HIS	89	O	B	3.23
PRO	124	O	A	SER	83	O	B	2.55
PRO	125	CG	A	PRO	86	CB	B	2.73
PRO	125	CA	A	GLU	84	O	B	3.13
ALA	126	N	A	GLU	84	O	B	3.06
ASP	150	O	A	PRO	82	CG	B	3.44
GLY	151	CA	A	PRO	82	CB	B	3.01
SER	338	O	A	PRO	8	CG	B	2.15
ILE	340	CD1	A	LEU	5	O	B	3.08
GLU	342	OE1	A	LEU	5	CB	B	1.52
GLU	342	OE2	A	SER	3	OG	B	2.87
GLU	342	OE1	A	ALA	6	N	B	3.20

Supplementary Table 1. Interacting residue analysis of *h*L3HYPDH experimental and predicted dimers. Labelled in green, interacting residues common to the experimental dimer (open conformation) and to the dimeric models predicted using template-based (Models 1 and 2) and *ab initio* (Models 3, 4 and 5) predictions. Unlabelled, unique interacting residues for the respective predicted models. A distance cut-off interval of 2.5-3.5 Å for the interacting residues has been applied. Highlighted in orange are the predicted interacting residue below the lower cut-off interval of 2.5 Å. The numbering of the dimeric models are as in Table 2 of the main text.
